# Supplementary material for: Dual-phase nanostructuring of layered metal oxides for high-performance aqueous rechargeable potassium ion microbatteries
Source: Nat Commun. 2019 Sep 20;10:4292. doi: 10.1038/s41467-019-12274-7 (PMC6754412; doi:10.1038/s41467-019-12274-7)
Supplement: Supplementary file 1 — Supplementary Information [file 41467_2019_12274_MOESM1_ESM.pdf]

Supplementary information

Dual-phase nanostructuring of layered metal oxides for high-performance aqueous rechargeable potassium ion microbatteries

Li et al

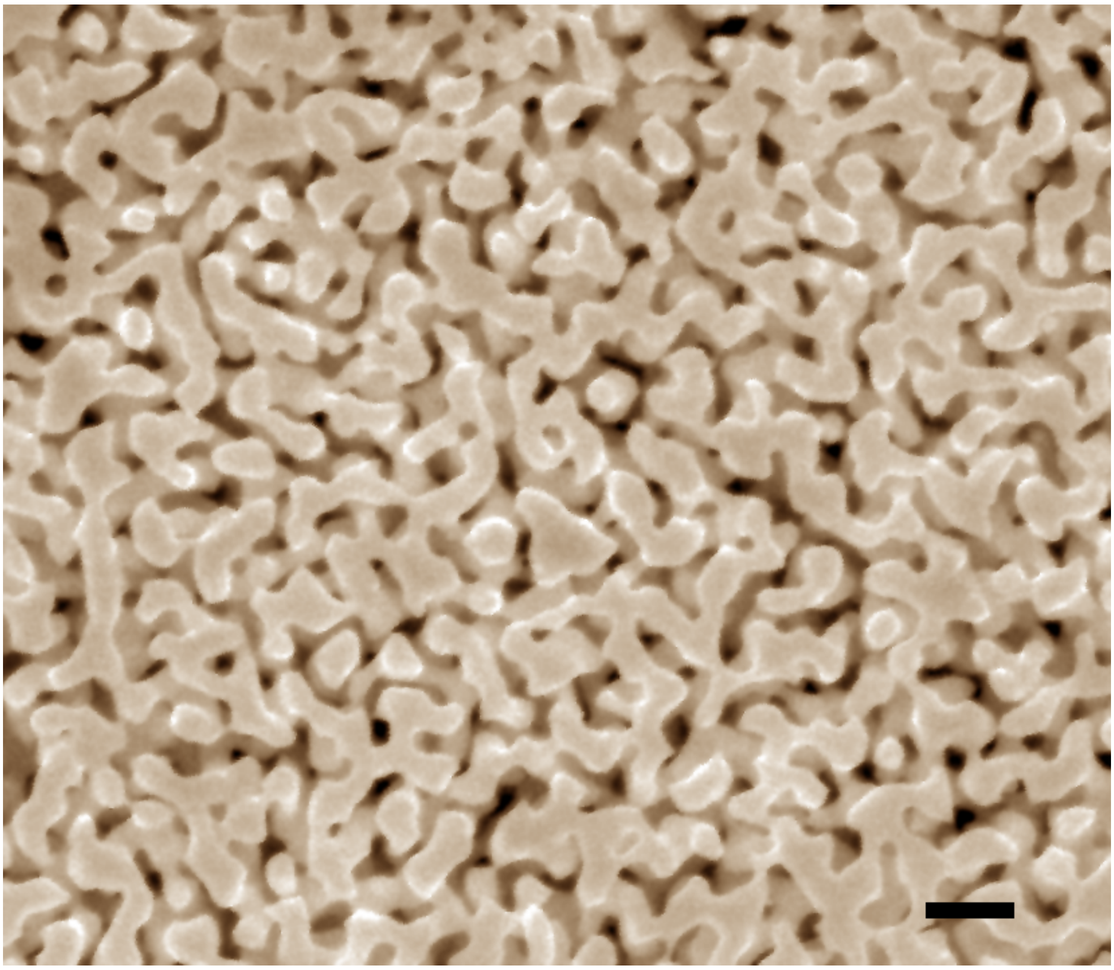

Supplementary Figure 1. Typical top-view SEM image for NP Au microelectrode. Scale bar, 200 nm.

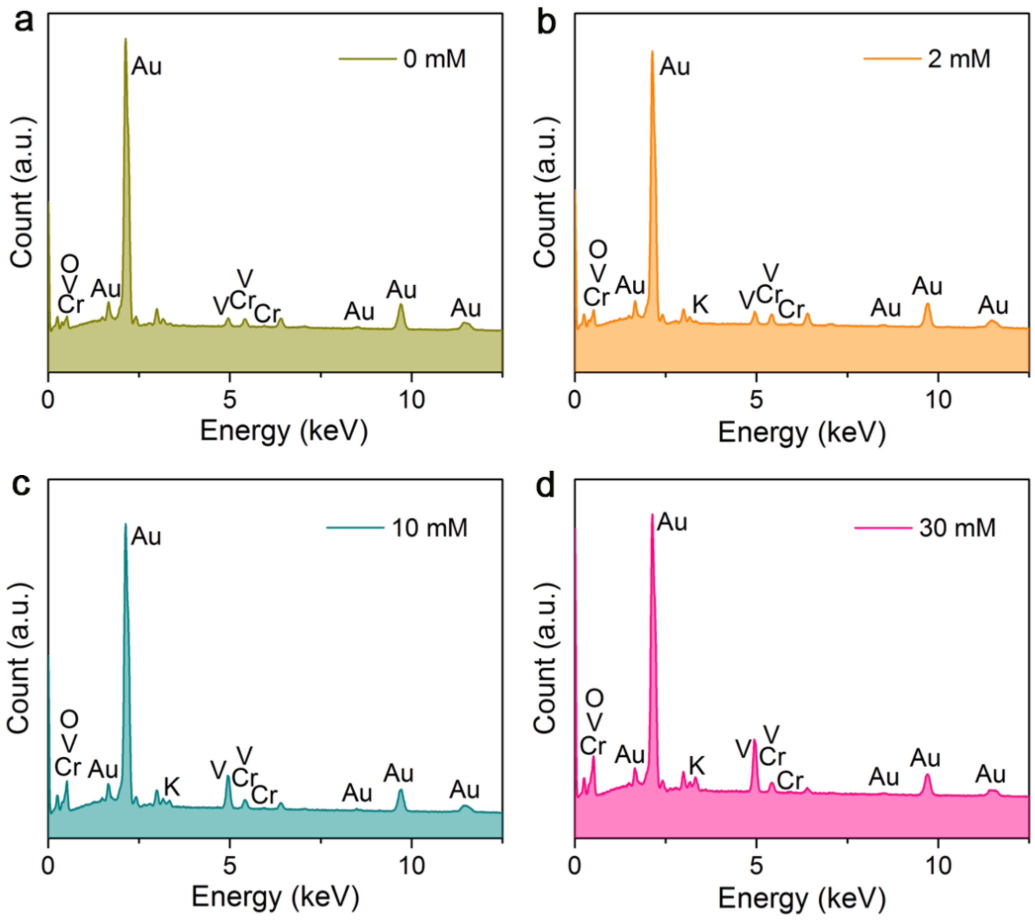

Supplementary Figure 2. Chemical analysis. EDS spectra of NP Au/$K_xV_2O_5$ specimens with the addition of various concentration of K_2SO_4 ranging from 0 to 30 mM for adjusting the x value from 0 to 0.25.

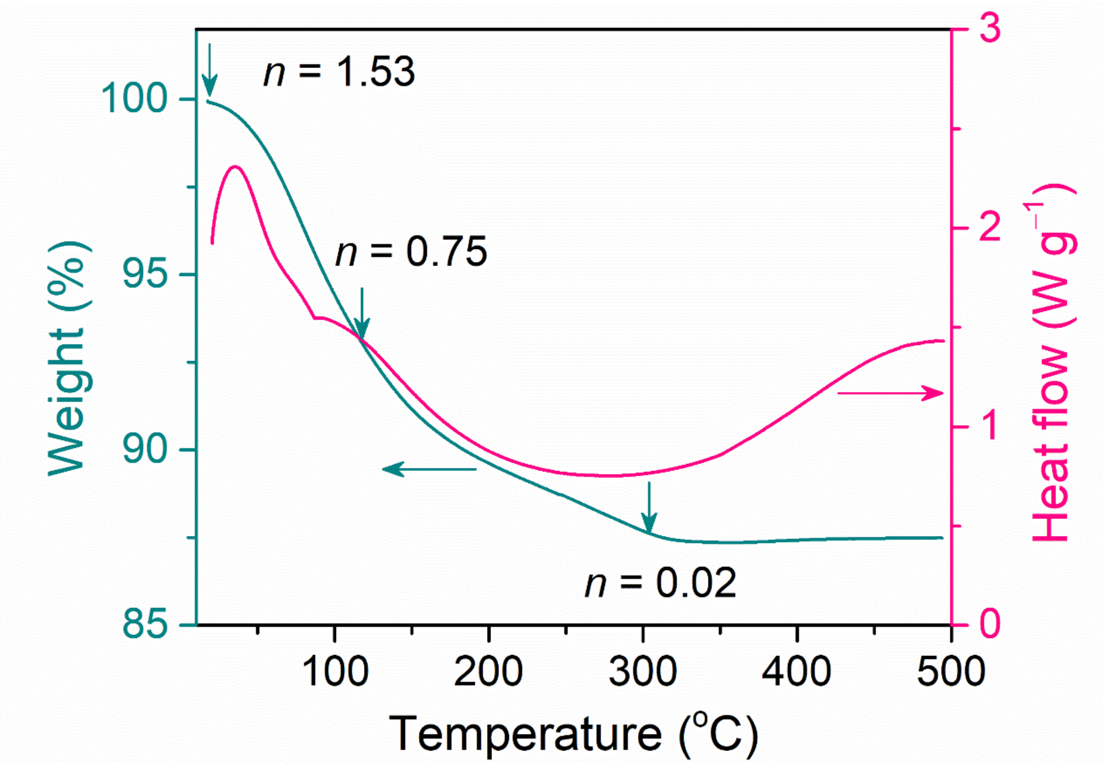

Supplementary Figure 3. Representative TGA and DSC curves for NP Au/$K_xV_2O_5$ (x = 0.25). TDA measurement reveals that ~1.53 mol of water exists in per mole of $K_xV_2O_5$ at room temperature. The weight evolution is characterized by a steep loss between room temperature and 110 °C (removing crystal water, to $K_xV_2O_5{\cdot}0.75H_2O$), followed by a more gradual weight loss up until 300 °C (removing structure water, to $K_xV_2O_5{\cdot}0.02H_2O$).

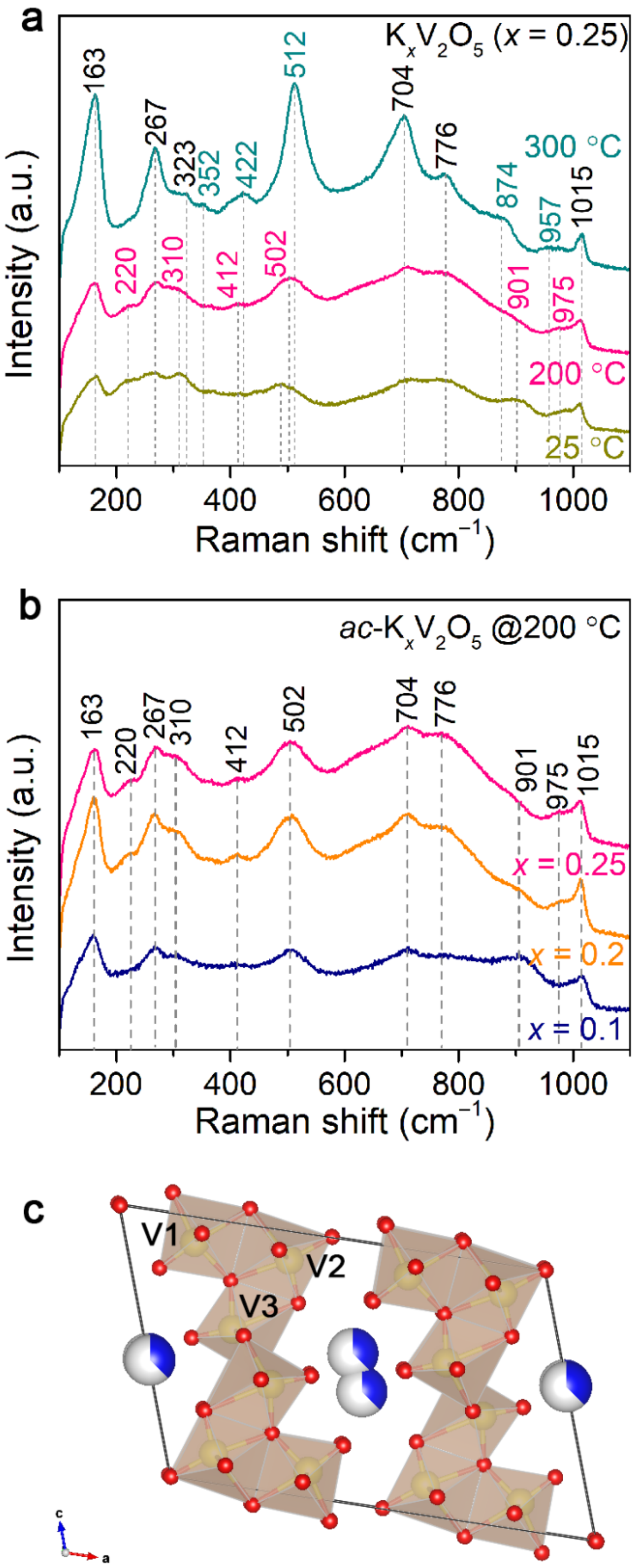

Supplementary Figure 4. **Raman spectra of NP Au/$K_xV_2O_5$ and atomic structure.** **a**, Raman spectra of NP Au/$K_xV_2O_5{\cdot}nH_2O$ (x = 0.25) that are annealed at 25, 200 and 300 °C for 12 h, respectively. **b**, Raman spectra of NP Au/$K_xV_2O_5{\cdot}nH_2O$ (x = 0.1, 0.2,

0.25) that are annealed at 200 °C for 12 h. **c**, Atomic schematic illustrating the crystalline structure of the *m*-$K_{0.25}V_2O_5$.

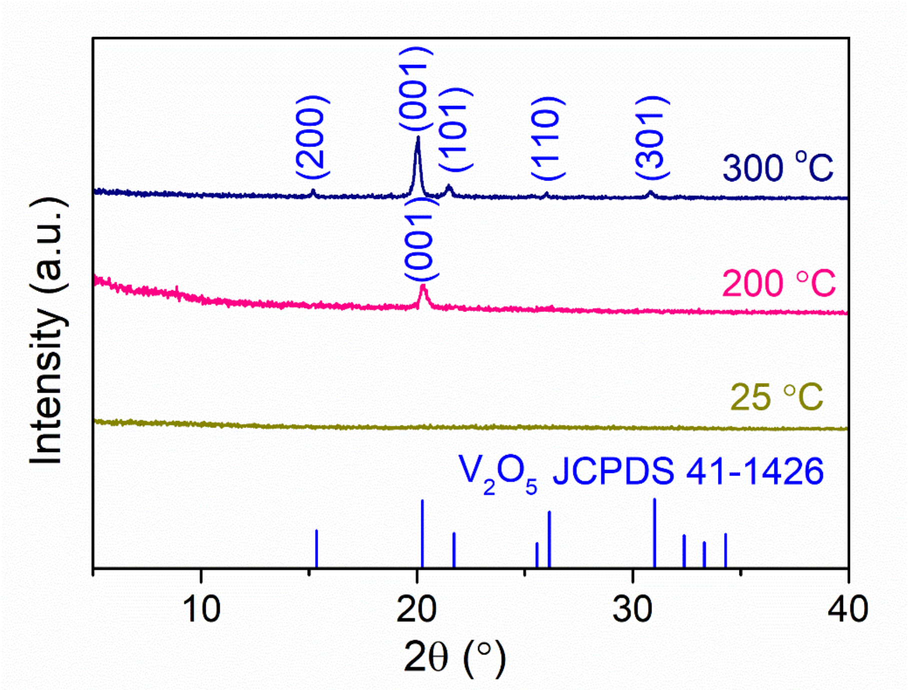

Supplementary Figure 5. XRD patterns of NP Au supported V_2O_5 that is annealed at 25, 200 and 300 °C.

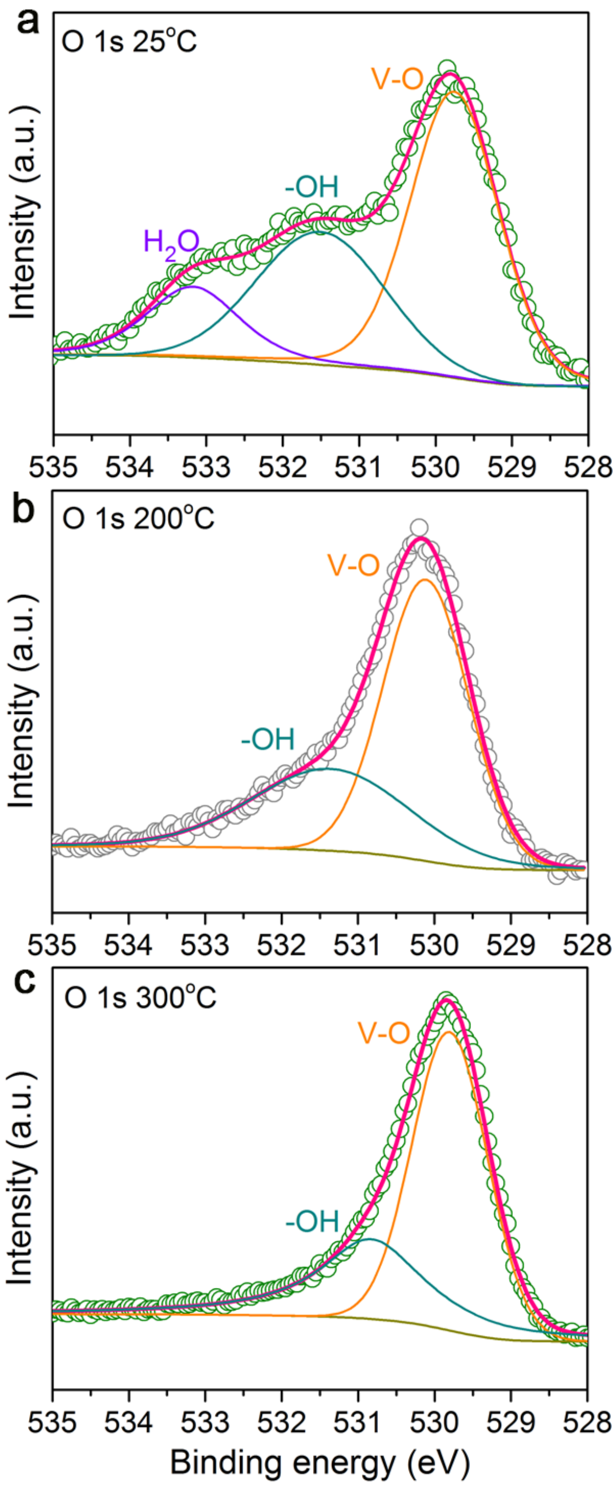

Supplementary Figure 6. **XPS spectra of O 1s**. O 1s XPS spectra for V_2O_5 annealed at temperatures of (a) 25 °C, (b) 200 °C and (c) 300 °C.

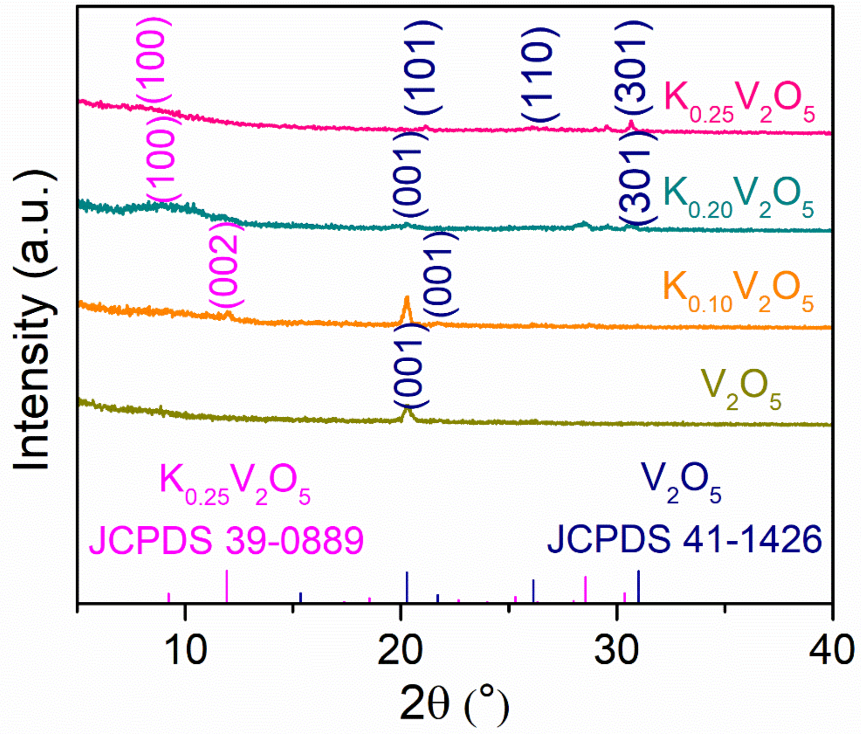

Supplementary Figure 7. XRD patterns of the constituent $K_xV_2O_5$ (x = 0, 0.1, 0.2 and 0.25) supported by NP Au microelectrodes, which are annealed at 200 °C in air.

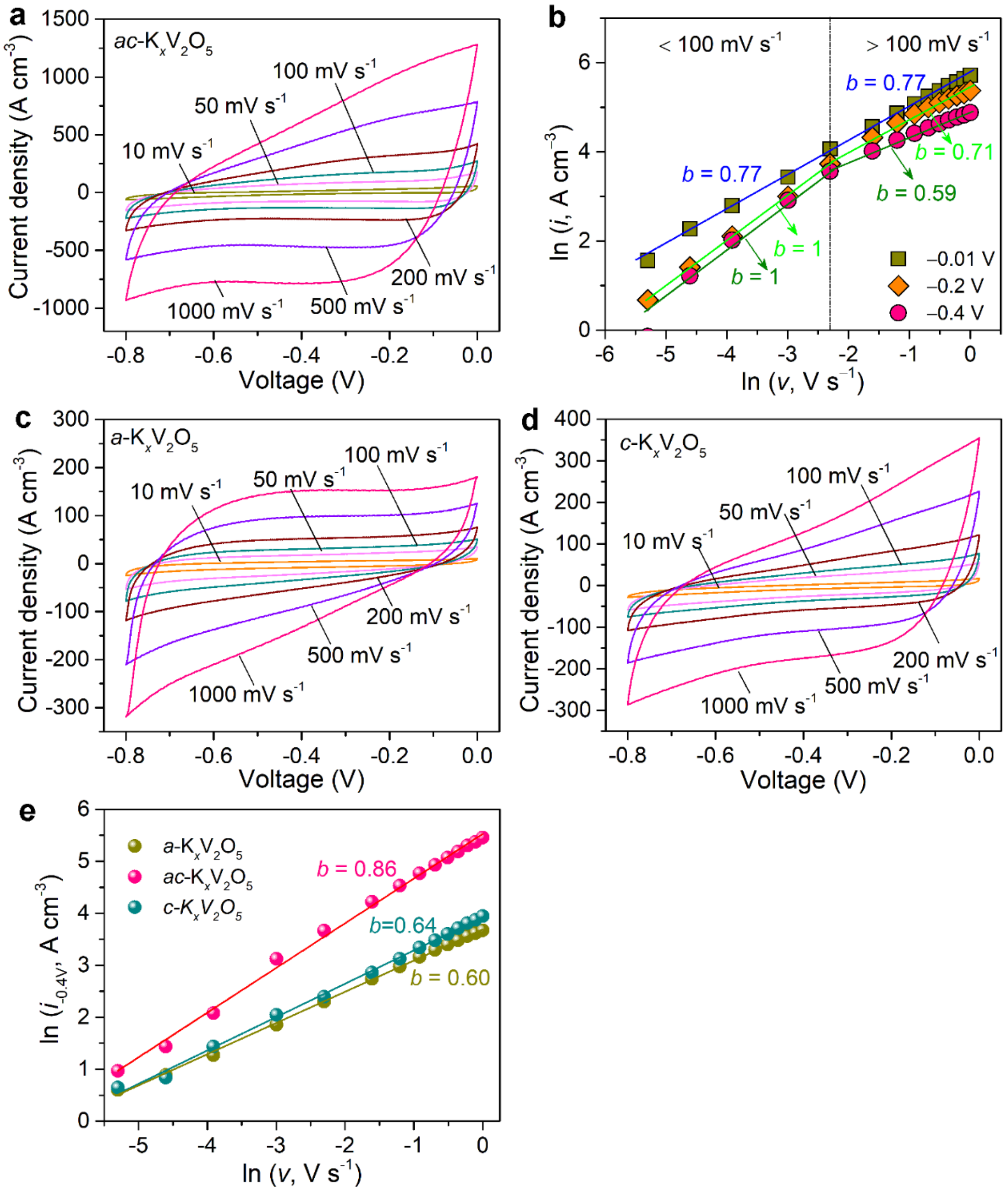

Supplementary Figure 8. **Electrochemical properties of hybrid electrodes. a**, Typical CV curves for NP Au/*ac*-$K_xV_2O_5$ (x = 0.25). **b**, Plot of ln(i) vs ln (v) for NP Au/*ac*-$K_xV_2O_5$ (x = 0.25) at the voltage of −0.01, −0.20 and −0.40 V. Therein, the b value is determined by assuming the power-law relationship between the current density and the scan rate, $i = av^b$. **c**,**d**, Typical CV curves for NP Au/*a*-$K_xV_2O_5$ (x = 0.25) (c) and NP Au/*c*-$K_xV_2O_5$ (x = 0.25) (d) at various scan rates ranging from 5 to 1000 mV s^{-1}. **d**, Plot of ln(i) vs ln (v) for NP Au/*a*-$K_xV_2O_5$ (x = 0.25), NP Au/*ac*-$K_xV_2O_5$ (x = 0.25) and NP Au/*c*-$K_xV_2O_5$ (x = 0.25) at the voltage of -0.4 V.

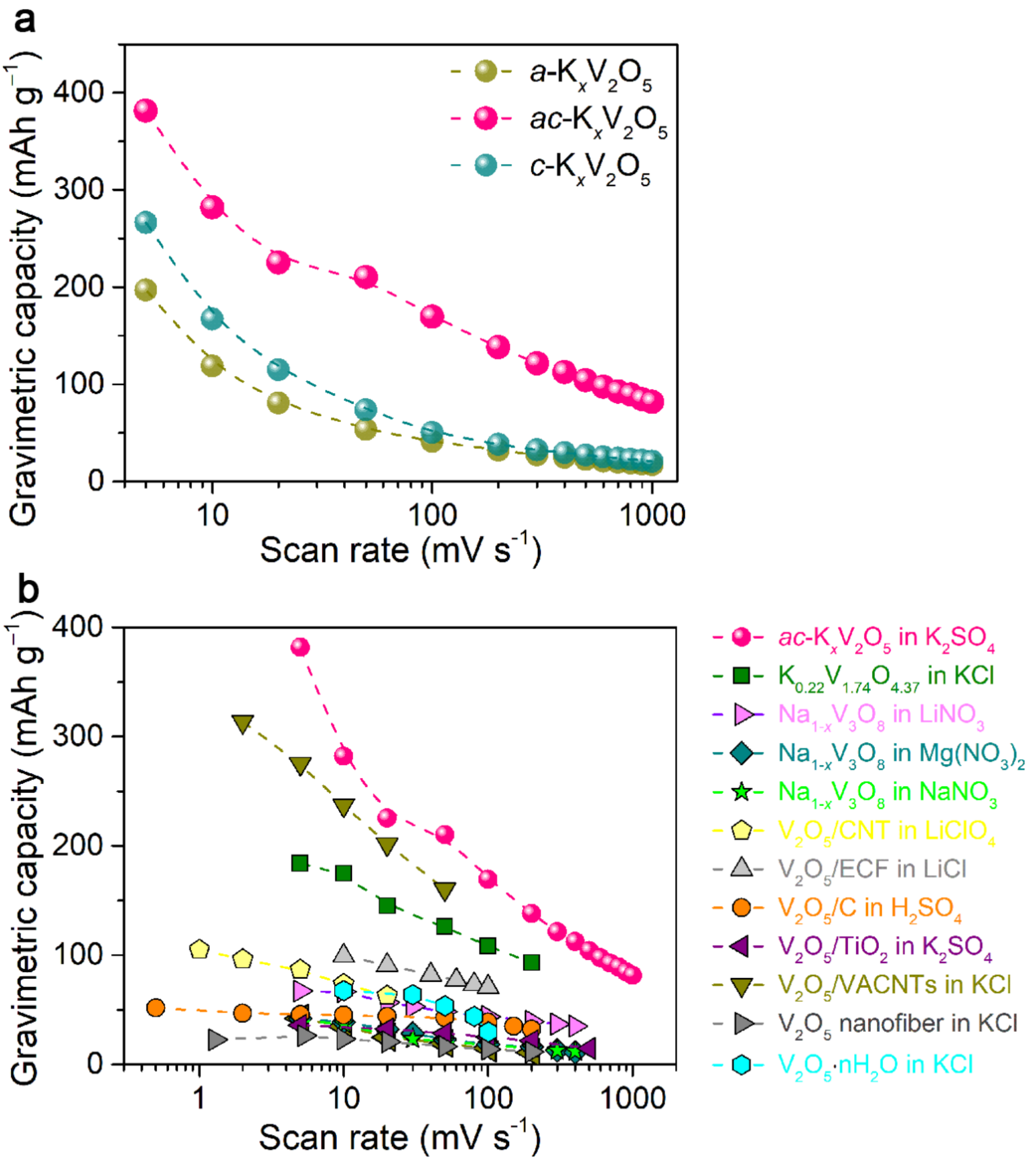

Supplementary Figure 9. **Comparison of gravimetric capacity. a**, Gravimetric capacity of the constituent *a*-$K_xV_2O_5$, *ac*-$K_xV_2O_5$ and *c*-$K_xV_2O_5$ (x = 0.25) at various scan rates. **b**, Comparison of gravimetric capacity of the constituent *ac*-$K_xV_2O_5$ at various scan rates, comparing with some of the best V_2O_5-based electrode materials for aqueous energy storage reported previously: such as $K_{0.22}V_{1.74}O_{4.37}$ in KCl,[1] $Na_{1-x}V_3O_8$ in $LiNO_3$, $Mg(NO_3)_2$ and $NaNO_3$,[2] V_2O_5/CNT in $LiClO_4$,[3] V_2O_5/ECF in LiCl,[4] C coated V_2O_5 in H_2SO_4,[5] V_2O_5/TiO_2 in K_2SO_4,[6] V_2O_5/VACNTs in KCl,[7] V_2O_5 nanofiber in KCl,[8] and $V_2O_5 \cdot nH_2O$ in KCl.[9]

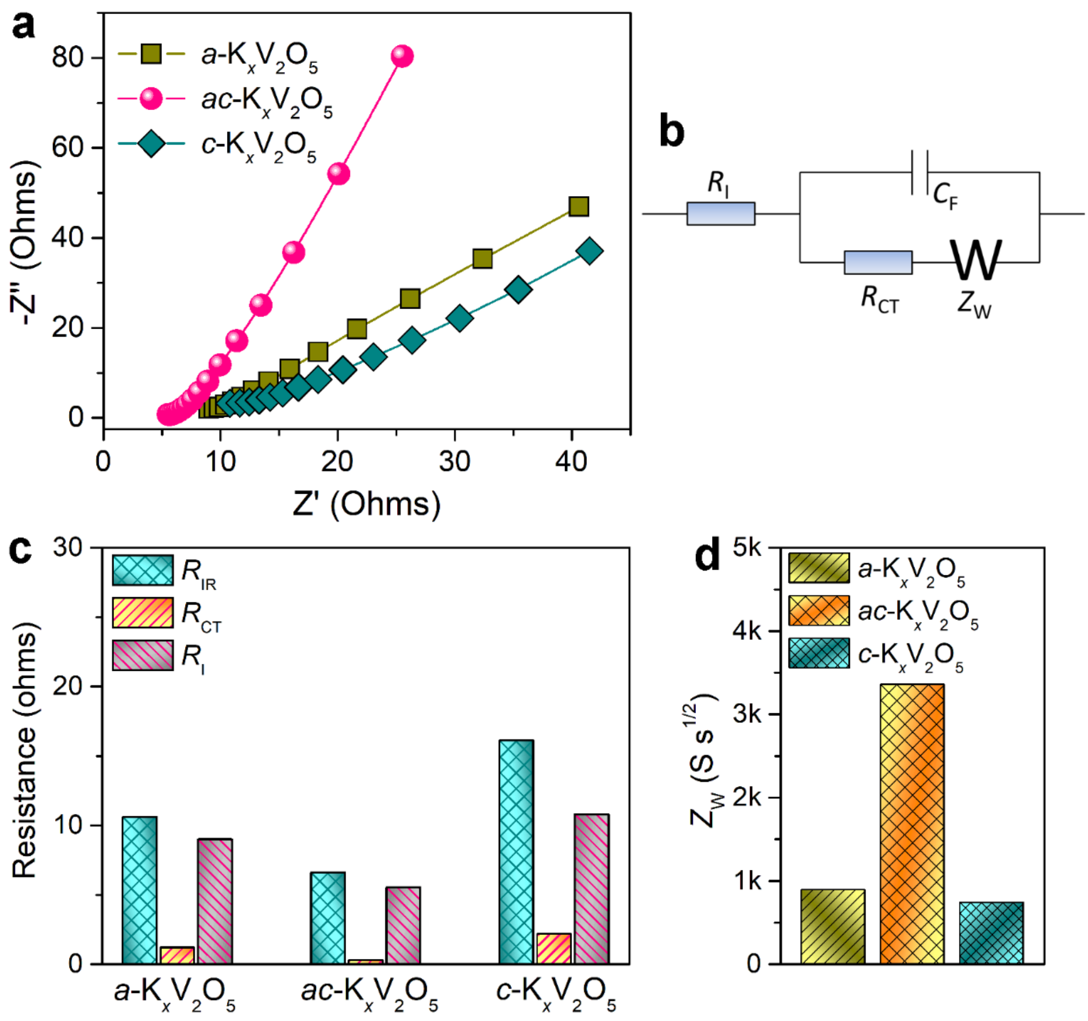

Supplementary Figure 10. **EIS spectra and resistance analysis. a**, Nyquist plot comparing electrochemical impedance spectra (EIS) of the NP Au/$K_xV_2O_5$ ($x = 0.25$) microelectrodes with different structures of *a*-$K_xV_2O_5$, *ac*-$K_xV_2O_5$, *c*-$K_xV_2O_5$. **b**, The electrical equivalent circuit used for fitting EIS. **c**, Comparisons the internal resistances, the intrinsic resistances (R_I) and the charge transfer resistances (R_{CT}) for NP Au/*a*-$K_xV_2O_5$, NP Au/*ac*-$K_xV_2O_5$ and NP Au/*c*-$K_xV_2O_5$ microelectrodes. **d**, Warburg resistance (Z_W) for NP Au/*a*-$K_xV_2O_5$, NP Au/*ac*-$K_xV_2O_5$ and NP Au/*c*-$K_xV_2O_5$ microelectrodes.

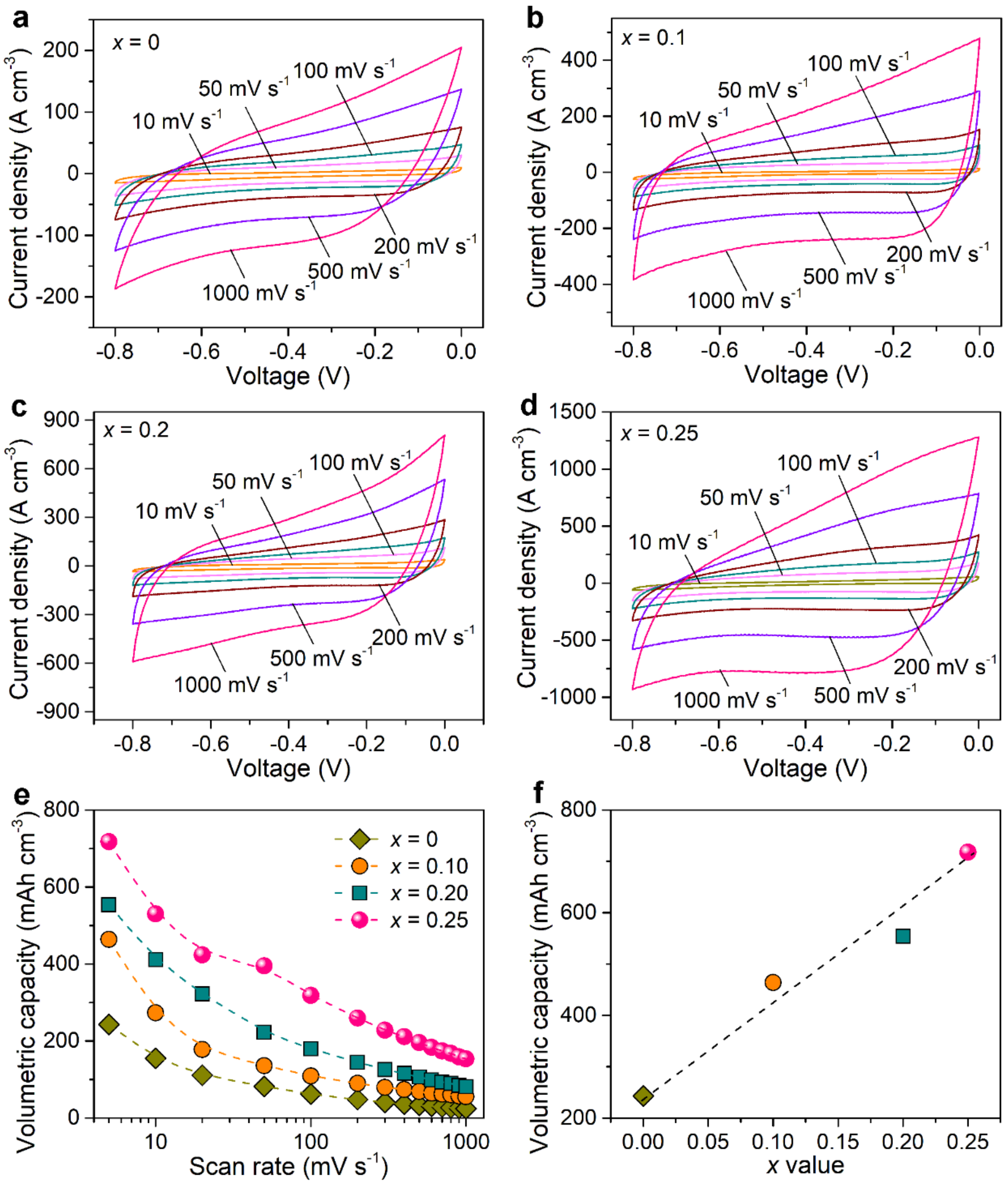

Supplementary Figure 11. **Electrochemical properties of NP Au/*ac*-$K_xV_2O_5$ microelectrodes.** CV curves of NP Au/*ac*-$K_xV_2O_5$ microelectrodes with, **a**, $x = 0$, **b**, $x = 0.1$, **c**, $x = 0.2$, and, **d**, $x = 0.25$, which are annealed their precursors at 200 °C, respectively. **e**, Comparison of volumetric capacities for NP Au/*ac*-$K_xV_2O_5$ microelectrodes with $x = 0$, 0.10, 0.20, 0.25. **f**, Volumetric capacities of NP Au/*ac*-$K_xV_2O_5$ microelectrodes at a scan rate of 5 mV s^{-1} as a function of K^+ concentration.

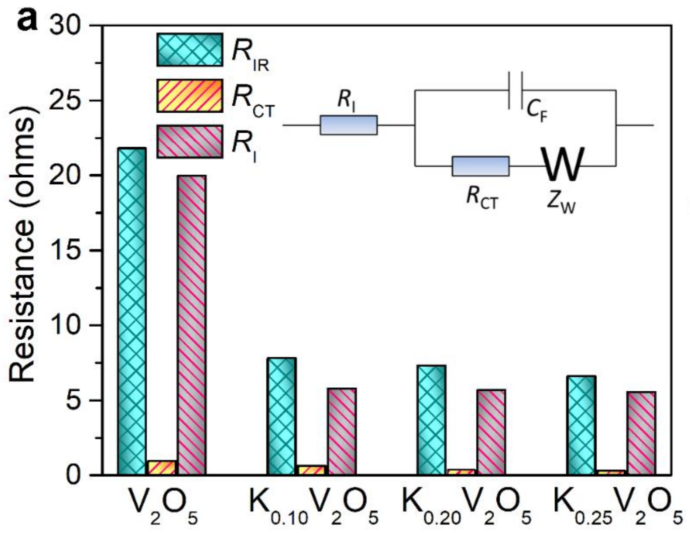

Supplementary Figure 12. Internal resistances of NP Au/*ac*-$K_xV_2O_5$ microelectrodes. **a**, Comparisons of the internal resistances, the intrinsic resistances (R_I) and the charge transfer resistances (R_{CT}) for NP Au/*ac*-$K_xV_2O_5$ (x = 0, 0.10, 0.20 and 0.25) microelectrodes, which are annealed at 200 °C for 12 h in air. Inset: The electrical equivalent circuit used for fitting EIS. **b**, Warburg resistance (Z_W) for NP Au/*ac*-$K_xV_2O_5$ (x = 0, 0.10, 0.20 and 0.25) microelectrodes.

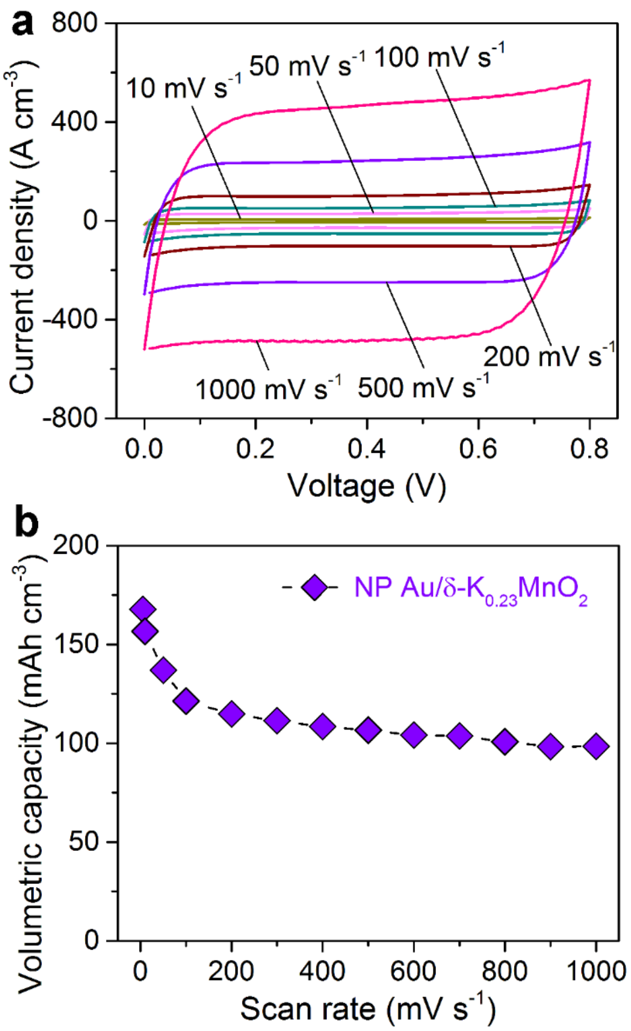

Supplementary Figure 13. **Electrochemical properties of NP Au/K_xMnO_2 cathode microelectrodes. a**, Representative CV curves of NP Au/K_xMnO_2 cathode microelectrodes at various scan rates from 5 to 1000 mV s^{-1}. **b**, Volumetric capacity of NP Au/K_xMnO_2 microelectrode as a function of scan rate.

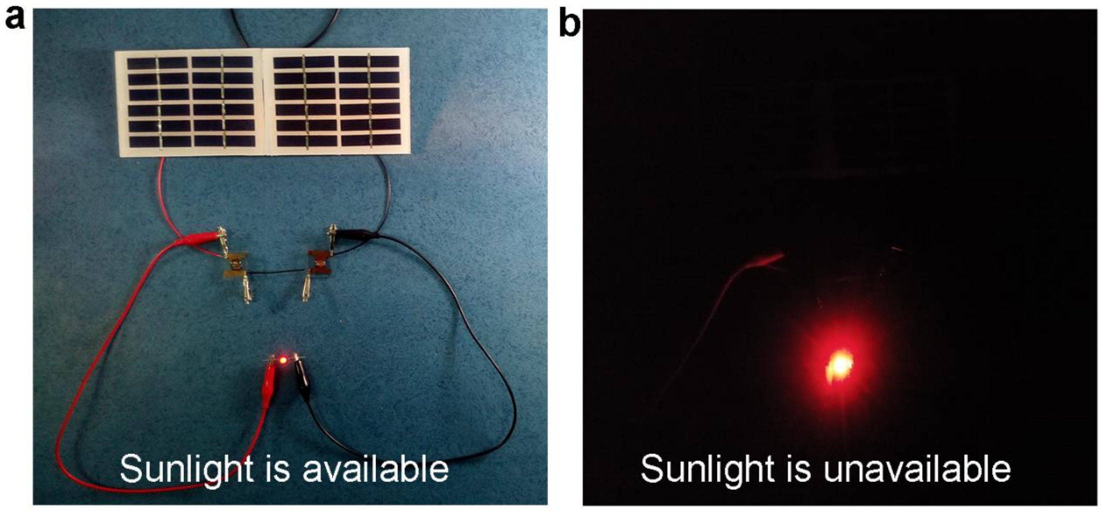

Supplementary Figure 14. **Demo for V-Mn AR-PIMBs integrated with solar cells.** **a**, **b**, Self-powered microsystems constructed with two series V-Mn AR-PIMBs and 2 V thin-film solar cells, LED light under sunlight (a) and dark (b).

Supplementary Table 1. Conditions for electrodeposititing a-$K_xV_2O_5$ on NP Au current microcollectors and their corresponding atomic ratio of 2K:V

Materials	Concentration of $VOSO_4$ (mM)	Concentration of K_2SO_4 (mM)	Atom ratio of 2K:V
a-V_2O_5	100	0	0
a-$K_{0.1}V_2O_5$	100	2	0.10
a-$K_{0.2}V_2O_5$	100	10	0.20
a-$K_{0.25}V_2O_5$	100	30	0.25

Supplementary references

1. Charles, D.S., Feygenson, M., Page, K., Neuefeind, J., Xu, W. & Teng, X. Structural water engaged disordered vanadium oxide nanosheets for high capacity aqueous potassium-ion storage. *Nat. Commun.* **8**, 15520 (2017).
2. Vujkovic, M., Paunkovic, B. S., Simatovic, I. S., Mitric, M., Sequeira, C. A. C., Mentus, S. Versatile insertion capability of $Na_{1.2}V_3O_8$ nanobelts in aqueous electrolyte solutions. *Electrochim. Acta* 147, 167-175 (2014).
3. Chen, Z., Augustyn, V., Wen, J., Zhang, Y. W., Shen, M. Q., Dunn, B., and Lu, Y. F. High-performance supercapacitors based on intertwined CNT/V_2O_5 nanowire nanocomposites. *Adv. Mater.* **23**, 791-795 (2011).
4. Li, L. L., Peng, S. J., Wu, H. B., Yu, L., Madhavi, S., and Lou, X. W. A flexible quasi-solid-state asymmetric electrochemical capacitor based on hierarchical porous V_2O_5 nanosheets on carbon nanofibers. *Adv. Energy Mater.* **5**, 1500753 (2015).
5. Elmouwahidi, A., Bailón-García, E., Pérez-Cadenas, A. F., Fernández-Sáez, N., and Carrasco-Marín, F. Development of vanadium-coated carbon microspheres: Electrochemical behavior as electrodes for supercapacitors. *Adv. Funct. Mater.* **28**, 1802337 (2018).
6. Samiee, M., and Luo, J. Pseudocapacitive properties of two-dimensional surface vanadia phases formed spontaneously on titania. *ACS Appl. Mater. Interfaces* **8**, 12871-12880 (2016).
7. Jiang, H. F., Cai, X. Y., Qian, Y., Zhang, C. Y., Zhou, L. J., Liu, W. L., Li, B. S., Lai, L. F., and Huang, W. V_2O_5 embedded in vertically aligned carbon nanotube arrays as free-standing electrodes for flexible supercapacitors. *J. Mater. Chem. A* **5**, 23727-23736 (2017).
8. Yeager, M. P., Du, W. X., Bishop, B., Sullivan, M., Xu, W. Q., Su, D., Senanayake, S. D., Hanson, J., and Teng, X. W. Storage of potassium ions in layered vanadium pentoxide nanofiber electrodes for aqueous pseudocapacitors. *ChemSusChem* **6**, 2231-2235 (2013).
9. Qian, A. N., Zhuo, K., Shin, M. S., Chun, W. W., Choi, B. N., and Chung, C. H. Surfactant effects on the morphology and pseudocapacitive behavior of $V_2O_5 \cdot H_2O$. *ChemSusChem* **8**, 2399–2406 (2015).
